# Adherence to the WHO’s Healthy Diet Indicator and Overall Cancer Risk in the EPIC-NL Cohort

**DOI:** 10.1371/journal.pone.0070535

**Published:** 2013-08-07

**Authors:** Nina E. Berentzen, Joline W. Beulens, Marieke P. Hoevenaar-Blom, Ellen Kampman, H. Bas Bueno-de-Mesquita, Dora Romaguera-Bosch, Petra H. M. Peeters, Anne M. May

**Affiliations:** 1 Julius Center for Health Sciences and Primary Care, University Medical Center Utrecht, Utrecht, The Netherlands; 2 Center for Nutrition, Prevention, and Health Services, National Institute for Public Health and the Environment (RIVM), Bilthoven, The Netherlands; 3 Division of Human Nutrition, Wageningen University, Wageningen, The Netherlands; 4 National Institute for Public Health and the Environment, Bilthoven, The Netherlands; 5 Department of Gastroenterology and Hepatology, University Medical Center Utrecht, Utrecht, The Netherlands; 6 Department of Epidemiology and Biostatistics, School of Public Health, Imperial College London, London, United Kingdom; 7 Department of Epidemiology and Public Health, Imperial College London, London, United Kingdom; The University of Texas M. D. Anderson Cancer Center, United States of America

## Abstract

**Background:**

A healthy dietary pattern defined by international recommendations of the World Health Organisation (WHO) has been shown to reduce overall mortality risk. It is unknown whether this healthy dietary pattern is associated with overall cancer incidence.

**Design:**

In total 35,355 men and women within the Dutch European Prospective Investigation into Cancer and Nutrition-cohort were followed for cancer occurrence. Diet was assessed through a validated food-frequency questionnaire. We computed a dietary score for all participants based on the seven WHO dietary guidelines for the prevention of chronic diseases (Healthy Diet Indicator (HDI)). We used the existing HDI score based on the 1990 WHO guidelines, and adapted it to meet with the 2002 WHO guidelines. Multivariate-adjusted Cox proportional hazards analysis was used to examine the association between adherence to the HDI and subsequent overall cancer risk.

**Results:**

A number of 3,007 new cancers were identified during a mean follow-up of 12.7 years. Adherence to the HDI was not associated with a reduced overall cancer risk. The hazard ratio (HR) of overall cancer associated with a one-point increment of the HDI was 0.96 (95% CI 0.89–1.03) in men, and 1.00 (95% CI 0.96–1.04) in women. Adherence to the HDI was not associated with smoking-related cancer ((HR men: 0.94 (95% CI 0.84–1.04); HR women: 1.00 (95% CI 0.94–1.07)), or alcohol-related cancer ((HR men: 1.02 (95% CI 0.87–1.20); HR women: 1.03 (95% CI 0.98–1.08)).

**Conclusions:**

Greater adherence to the WHO’s Healthy Diet Indicator, a dietary pattern for prevention of chronic diseases, was not associated with reduced overall, smoking-related or alcohol-related cancer risk in men or women.

## Introduction

The Netherlands has the 12th highest cancer rates in the world. Every year 286.8 people out of every 100,000 develop cancer [1] and the disease accounts for nearly one third of total annual mortality in the Netherlands [2]. Dietary habits are recognized to be important modifiable factors influencing cancer risk [3], [4] and have been estimated, together with overweight/obesity and physical activity, to account for approximately 35–38% of 12 common cancers in high-income countries, according to the World Cancer Research Fund/American Institute for Cancer Research (WCRF/AICR) [5]. Dietary patterns examine effects of overall diet and allow for underlying synergistic effects between the individual dietary components [6]. Most of the studies that relate dietary patterns to health outcomes use either a priori (researcher-driven) diet scores, or a posteriori (data-driven) scores derived from factor- or cluster analysis. A priori dietary scores can be further grouped into three categories; (a) scores that assess dietary variety or diversity, (b) scores that assess concordance with dietary guidelines and (c) scores that assess specific dietary patterns (e.g. the Mediterranean diet) [7]. The Mediterranean dietary pattern has been found to reduce risk for cancer morbidity and mortality for some countries (especially the Mediterranean countries), but not for other, more Northern countries such as the Netherlands [8], [9].

In 1990, the World Health Organisation (WHO) published international dietary guidelines for prevention of chronic diseases. Successively, the Healthy Diet Indicator (HDI) was developed by Huijbregts et al. [10] to quantify adherence to these guidelines. Previous studies have related the HDI to overall- and cancer-specific mortality. The HDI was found to be inversely related to all-cause mortality in elderly men of three European countries (RR for high versus low HDI adherence: 0.87, 95% CI: 0.77–0.98) [10]. This study also found risk of death from cardiovascular disease and cancer to be respectively 18% and 15% lower in the highest HDI group than in the lowest group, but specific estimates were not provided. In another cohort of elderly European men and women, a higher HDI was related to lower all-cause mortality (HR: 0.89 with 95% CI: 0.81–0.98), however, cancer mortality was not investigated [11]. In addition, the HDI was studied in relation to breast cancer risk in British women; however, no association was found (HR for maximal adherence to the HDI compared with minimal adherence: 0.94 with 95% CI: 0.67–1.32) [12].

As far as we know, no prospective study has related adherence to this dietary pattern to the occurrence of overall cancer. We aimed to investigate the association between adherence to the HDI and risk of overall cancer incidence, as well as alcohol- and smoking-related cancer. We examined associations separately for men and women participating in the Dutch part of the European Prospective Investigation into Cancer and Nutrition (EPIC-NL) cohort study.

## Subjects and Methods

### Study Population

The EPIC-NL study consists of the two Dutch contributions to the EPIC cohort: Prospect and MORGEN cohorts [13]. The study design has been described elsewhere [14]. In brief, Prospect is a prospective cohort study of 17,357 women, aged 49–70, who participated in breast cancer screening between 1993 and 1997 [15]. The MORGEN cohort consists of 22,654 men and women aged 20–65 years recruited from three Dutch cities (Amsterdam, Doetinchem, and Maastricht) between 1993 and 1997 [16]. In total, there were 40,011 participants in the EPIC-NL cohort. All participants provided written informed consent before study inclusion. The study complies with the Declaration of Helsinki and was approved by the institutional board of the University Medical Center Utrecht (Prospect) and the Medical Ethical Committee of TNO Nutrition and Food Research (MORGEN).

The present analysis was restricted to participants with no prior history of cancer and with complete dietary data. Initially, 40,011 participants were available out of which 39,793 participants had complete dietary data. Participants with prevalent cancer or with missing data regarding history of cancer (n = 1688) were excluded. Participants who gave no permission for linkage with vital status registries were excluded (n = 2028), as well as participants without follow-up data (n = 391). Participants who reported unlikely energy intakes (n = 331) were excluded (those in the top 0.5% and bottom 0.5% of the ratio of self-reported energy intake to basal metabolic rate). In total, 35,355 participants were included in the final study population (9,188 men and 26,167 women). The analyses were restricted to first incident cancers.

### Healthy Diet Indicator

To quantify adherence to the WHO’s guidelines for prevention of chronic diseases we used the Healthy Diet Indicator, which incorporated 7 WHO recommendations regarding nutrients or food groups [17]. Daily dietary intake was obtained at recruitment from a food-frequency questionnaire (FFQ) containing questions on the usual frequency of consumption of 79 main foods during the year preceding recruitment. This questionnaire allows the estimation of the average daily consumption of 178 foods. The FFQ has been validated against twelve 24-h recalls, administered once a month for one year [18], [19]. Pearson correlation coefficients were 0.61 (men) and 0.63 (women) for fat, 0.71 (men) and 0.67 (women) for protein, 0.74 (men) and 0.76 (women) for carbohydrate, and 0.61 (men) and 0.74 (women) for fibre. Macro- and micronutrients values of reported foods (expressed per 100 grams edible portion) were obtained from national tables compiled by the Dutch Food Composition Database (NEVO). The HDI was originally created by Huijbregts et al. according to WHO recommendations of 1990 [10]. The WHO provided updated guidelines in 2002 and we adapted the HDI accordingly [20]. Detailed information on the operationalization of the HDI is shown in Table 1. Seven food groups and nutrients were included in the updated HDI: saturated fatty acids; polyunsaturated fatty acids; cholesterol; protein; dietary fibre; fruits and vegetables; and free sugars. In concordance with the updated WHO guidelines, three former HDI components were omitted in the updated HDI: ‘monosaccharides and disaccharides’, ‘complex carbohydrates’ and ‘pulses, nuts and seeds’. Also, the component ‘free sugars’ (including monosaccharides, disaccharides, and sugars from honey, syrups and fruit juices) was added to the updated HDI. We excluded the component salt from the HDI because we did not have valid information: previously Huijbregts et al. also excluded this recommendation since only sodium content in foods was available but it was unknown how much salt was added during preparation of meals and at the table.

**Table 1:** Composition of the Healthy Diet Indicator^1^ (HDI) used in analyses of cancer, based on the WHO's dietary guidelines for the prevention of chronic diseases.

	Scoring criteria	
Nutrient or food group (daily intake)	Criteria for 1 point	Criteria for 0 points
Saturated fatty acids (en%)^2^	<10	≥10
Polyunsaturated fatty acids (en%)^2^	6–10	<6 or >10
Cholesterol (mg)	<300	≥300
Protein (en%)^2^	10–15	<10 or >15
Dietary fibre (g)	>25	≤25
Fruits and vegetables (excluding potatoes) (g)	≥400	<400
Free sugars (en%)^2^	<10	≥10

1 HDI range was 0–7 points. Tertiles of adherence to the HDI was; T1: <3 points, T2∶3 points, T3: >3 points.

2 (en%) refers to the percentage of total energy intake excluding alcohol.

Abbreviations: mg, milligrams, g, grams, en%, energy percentage.

A dichotomous variable was generated for each component of the HDI. If a person’s intake was within the recommended range according to WHO’s guidelines this variable was coded as 1; otherwise it was coded as 0. The HDI was the sum of all these dichotomous variables and had a range of 0–7 points.

### Ascertainment of Cancer Events

During follow-up, participants were followed for disease occurrence and cancer cases were identified by annual linkage to the Netherlands Cancer Registry. This registry identifies incident cancer cases through pathology records and is 95% complete since 1989. Follow-up for cancer incidence and vital status was complete until December 31, 2008. Prevalent cases of cancer were also identified through linkage with the cancer registry and by self-report using the baseline general questionnaire [14]. Smoking-related cancer was defined as cancer of the lung, kidney, upper aero-digestive tract, liver, stomach, pancreas, bladder and colorectum [21], [22]. Alcohol-related cancer included cancer of the upper aero-digestive tract, breast, liver, and colorectum [23].

### Covariates

At baseline, participants completed a lifestyle questionnaire including questions on demographics, presence of chronic diseases, and risk factors for chronic diseases. Participants returned the questionnaire when coming for a physical examination, and completeness of the questionnaire was discussed. During the physical examination height and weight were measured, and body mass index (BMI; kg/m^2^), was calculated. Physical activity was assessed using the EPIC physical activity questionnaire [24] and categorized according to the validated Cambridge Physical Activity Index (inactive, moderately inactive, moderately active, active) [25], [26]. Because data on physical activity was not available for 14% of the EPIC-NL cohort, these missing values were imputed using single linear regression modelling [27]. Smoking status was categorized as never, former, current smoking, and current smokers were further categorized into categories of average number (1, 2–10, 10–20, >20) of cigarettes per day. Education was categorized as low (primary to completing intermediate vocational education) intermediate (high secondary education), and high (high vocational education or university).

### Statistical Analysis

Simple tabulations were made for sociodemographic data by sex and by tertiles of the HDI. Multivariate Cox proportional hazards regression models were fitted to estimate cancer hazard ratios (HRs) and corresponding 95% confidence intervals (CIs). The time variable was the interval between date of recruitment to date of cancer diagnosis or censoring (death, lost to follow-up or end of follow-up (December 2008)), whichever occurred first.

HRs were calculated for overall cancer, smoking-related cancer and alcohol-related cancer. Results were computed for men and women separately because of differences in cancer types and confounders. All models were stratified by cohort (Morgen or Prospect).

The HDI was analysed as a continuous variable (per 1-unit increase of the HDI) and in three groups of approximately equal numbers (HDI = 0–2, 3, and 4–7) with the first tertile (least healthy HDI) as the reference category. P-values for linear trend across the tertiles were calculated by including the categorical HDI as a continuous variable in the model.

Multivariate analyses were adjusted for age at baseline (years), BMI, smoking status, total energy intake excluding energy from alcohol (kcal/day), alcohol intake (g/day), physical activity level and educational level. Analyses in women were additionally adjusted for menopausal status (pre-, peri-, and postmenopausal; surgical menopause; or missing). Parity, breastfeeding, hormone-replacement therapy, and marital status were not included in the model since these variables were not confounders in the association between HDI and overall or alcohol-related cancer. Possible modifying effects of sex, BMI and smoking status were investigated by adding interaction terms (with the continuous HDI variable) to the statistical model.

We estimated the individual association of each component of the HDI with overall cancer risk, adjusting for all six other components of the score and for the covariates mentioned before.To examine whether associations would be different for participants who developed cancer early or late during follow-up relative to the baseline dietary measurement we repeated the main analysis in follow-up periods of <5 years, 5–10 years and >10 years.

Additionally, we computed models excluding participants with less than two years of follow-up to prevent that dietary habits had changed in response to early symptoms of the yet undiagnosed cancer. We also investigated whether associations were different for the updated HDI (according to WHO 2002 guidelines) as compared to the former HDI used by Huijbregts et al. (according to 1990 WHO guidelines). For that aim we repeated the main analysis of overall cancer using Huijbregts’ compilation of the HDI [10]. All analyses were performed using SAS, version 9.2 (SAS Institute, Cary, NC).

## Results

During 12.7 years of follow-up, 586 men and 2,421 women were diagnosed with cancer. Due to the population selection for EPIC-NL, mean age at baseline in tertiles of the HDI varied between 42 and 44 years for men and between 51 and 52 years for women (Table 2). The percentage current smokers ranged from 28–40% in men; and from 22–30% in women between HDI tertiles, while alcohol intake ranged from 16–20 g/day in men; and from 8–9 g/day in women between HDI tertiles. A higher adherence to the HDI was observed in participants with higher education level, higher physical activity levels and among never and former smokers. Men and women with high adherence to the HDI also had a lower BMI and lower use of alcohol, compared with participants with low adherence to the HDI.

**Table 2:** Baseline characteristics and number of incident cancers in the EPIC-NL cohort according to tertiles of adherence to the Healthy Diet Indicator (HDI).

			Adherence to HDI^1^					
			Men (n = 9188)			Women (n = 26167)		
Baseline characteristic		All participants	Tertile 1^2^	Tertile 2^2^	Tertile 3^2^	Tertile 1^2^	Tertile 2^2^	Tertile 3^2^
*N* (%)		35355 (100.0)	2433 (26.5)	3173 (34.5)	3582 (39.0)	6917 (26.4)	9577 (36.6)	9673 (37.0)
Age (years; mean, s.d.)		49.2 (11.9)	44.1 (11.0)	43.6 (11.0)	42.4 (11.1)	52.0 (11.3)	51.2 (11.3)	50.8 (11.8)
Body Mass Index (kg/m^2^; mean, s.d.)		25.7 (4.0)	26.1 (3.6)	25.9 (3.5)	25.5 (3.5)	25.8 (4.2)	25.7 (4.2)	25.5 (4.1)
Energy intake (kcal/day; mean, s.d.)		1977 (590)	2357 (652)	2427 (693)	2556 (601)	1733 (449)	1745 (444)	1925 (460)
Alcohol intake (g/day; mean, s.d.)		11.0 (15.3)	20.3 (23.6)	18.5 (20.1)	15.9 (17.9)	8.7 (12.6)	9.2 (12.7)	7.8 (11.2)
Smoking status *(N, %)*								
Never		13508 (39.2)	653 (27.7)	915 (29.8)	1224 (35.7)	2796 (41.1)	3745 (40.0)	4175 (44.1)
Former		11293 (32.7)	765 (32.5)	1087 (35.5)	1248 (36.4)	1995 (29.3)	2978 (31.8)	3220 (34.0)
Current		9687 (28.1)	939 (39.8)	1064 (34.7)	960 (28.0)	2016 (29.6)	2637 (28.2)	2071 (21.9)
Education *(N, %)*								
Low		24440 (69.5)	1606 (66.3)	1959 (62.0)	22120 (59.3)	5226 (76.1)	6921 (72.7)	6608 (68.7)
Middle		3533 (10.0)	274 (10.9)	341 (10.8)	373 (10.4)	649 (9.5)	932 (9.8)	974 (10.1)
High		7200 (20.5)	551 (22.8)	862 (27.3)	1085 (30.3)	989 (14.4)	1674 (17.6)	2039 (21.2)
Physical activity *(N, %)*								
Inactive		3208 (9.1)	396 (16.3)	396 (12.5)	313 (8.7)	699 (10.1)	741 (7.7)	663 (6.9)
Moderately inactive		10184 (28.8)	783 (32.2)	996 (31.4)	1033 (28.8)	2019 (29.2)	2841 (29.7)	2512 (26.0)
Moderately active		9815 (27.8)	646 (26.6)	912 (28.7)	1077 (30.1)	1869 (27.0)	2657 (27.7)	2654 (27.4)
Active		12148 (34.4)	608 (25.0)	869 (27.4)	1159 (32.4)	2330 (33.7)	3338 (34.9)	3844 (39.7)
Menopausal status *(N, %)*								
Premenopausal		8273 (31.6)	–	–	–	2004 (29.0)	3048 (31.8)	3221 (33.3)
Postmenopausal		12441 (47.5)	–	–	–	3425 (49.5)	4472 (46.7)	4544 (47.0)
Cancer cases *(N, %)*								
Overall cancer		3007 (8.5)	167 (6.9)	225 (7.1)	194 (5.4)	688 (10.0)	864 (9.0)	869 (9.0)
Smoking-related cancer		1032 (3.1)	93 (3.9)	98 (3.2)	89 (2.6)	209 (3.2)	290 (3.2)	253 (2.8)
Alcohol-related cancer		1413 (4.2)	37 (1.6)	45 (1.5)	45 (1.3)	346 (5.3)	459 (5.0)	481 (5.2)

1 HDI (range 0–7 points) included 7 components: saturated fatty acids, polyunsaturated fatty acids, cholesterol, protein, fibre, fruits and vegetables and free sugars.

2 HDI tertiles: T1: <3 points; T2∶3 points; T3: >3 points.

Adherence to the HDI was not significantly associated with a reduction in overall cancer risk (Table 3). The hazard ratio (HR) of overall cancer associated with a 1-point increment of the HDI was 0.99 (95% CI 0.96–1.02) for the total cohort; 0.96 (95% CI 0.89–1.03) for men; and 1.00 (95% CI 0.96–1.04) for women. Tertile-specific HRs for men were 1.12 (95% CI 0.91–1.37) and 0.93 (95% CI 0.75–1.15), for a HDI of 3 (tertile 2) and 4–7 (tertile 3) compared with 0–3 (tertile 1) (*P* for linear trend = .46). For women, HRs were 0.93 (95% CI 0.84–1.03) for the second, and 0.98 (95% CI 0.88–1.08) for the third tertile of adherence to thze HDI (*P* for linear trend = .67).

**Table 3:** Multivariable hazards ratios (HRs) and 95% CIs of cancer according to tertiles of adherence to the Healthy Diet Indicator (HDI) in the EPIC-NL cohort.

	HR (95% CI)^1^					
Group of cancers	Tertile 1	Tertile 2		Tertile 3	Continuous (per 1-point increment)	P for trend^5^
Overall cancer						
All participants	1 (referent)		0.97 (0.89–1.06)	0.97 (0.88–1.06)	0.99 (0.96–1.02)	.53
Men	1 (referent)		1.12 (0.91–1.37)	0.93 (0.75–1.15)	0.96 (0.89–1.03)	.46
Women^2^	1 (referent)		0.93 (0.84–1.03)	0.98 (0.88–1.08)	1.00 (0.96–1.04)	.67
Smoking-related cancer^3^						
Men	1 (referent)		0.90 (0.68–1.20)	0.83 (0.62–1.12)	0.94 (0.84–1.04)	.23
Women^2^	1 (referent)		1.07 (0.90–1.28)	1.03 (0.85–1.24)	1.00 (0.94–1.07)	.78
Alcohol-related cancer^4^						
Men	1 (referent)		1.00 (0.64–1.54)	0.97 (0.62–1.52)	1.02 (0.87–1.20)	.90
Women^2^	1 (referent)		0.97 (0.84–1.11)	1.05 (0.91–1.21)	1.03 (0.98–1.08)	.46

1 All models were stratified by sex and cohort, and adjusted for age at baseline, body mass index, smoking status, education, physical activity, energy intake without energy from alcohol, and alcohol intake.

2 Models in women were additionally adjusted for menopausal status.

3 Smoking-related cancer included cancer of the lung, kidney, upper aero-digestive tract, stomach, pancreas, bladder, liver, and colorectal.

4 Alcohol-related cancer included cancer of the upper aero-digestive tract, breast, liver, and colorectal.

5 P for trend values were calculated using two-sided test for linear trend, treating the HDI categories as a continuous variable.

Adherence to the HDI was not significantly associated with risk of smoking-related and alcohol-related cancer (Table 3). For smoking-related cancers, the HR per 1-point increment of the HDI was 0.94 (95% CI 0.84–1.04) for men and 1.00 (95% CI 0.94–1.07) for women. For alcohol-related cancers, the HR per 1-point increment of the HDI was 1.02 (95% CI 0.87–1.20) for men and 1.03 (95% CI 0.98–1.08) for women.

No statistically significant interaction was found between the HDI and sex (*P* for interaction = .22), BMI (*P* = .83), or smoking status (*P* = .89) on overall cancer (results not shown).

When examining possible associations between the seven components of the HDI and overall cancer risk (Table 4), we found a borderline statistically significant increase in cancer risk with saturated fat intake. The HR for overall cancer associated with an increment in daily saturated fat intake of 3 percent of total energy intake was 1.06 (95% CI 1.00–1.11). No statistically significant associations were observed for the other components. Associations for overall cancer were comparable for all follow-up periods. For cancers occurring within 5 years the HR for overall cancer per one-point increment of the HDI was 0.99 (95% CI 0.93–1.06) (men and women combined); for cancers occurring within 5–10 years the HR was 1.02 (95% CI 0.97–1.08); for cancers occurring >10 years the HR was 1.00 (95% CI 0.95–1.06).

**Table 4:** HR and 95% CI for overall cancer associated with increments in the components of the Healthy Diet Indicator (men and women combined).

HDI component	Mean (s.d.) consumption	Increment^1^	HR (95% CI)^2^
Saturated fatty acids (en%/day)^3^	14.6 (2.6)	3	1.06 (1.00–1.11)
Polyunsaturated fatty acids (en%/day)^3^	6.7 (1.8)	2	1.00 (0.97–1.05)
Cholesterol (mg/day)	225.4 (85.6)	86	1.00 (0.94–1.07)
Protein (en%/day)^3^	16.1 (2.4)	2	1.00 (0.97–1.05)
Dietary fibre (g/day)	23.8 (6.6)	7	1.01 (0.94–1.09)
Fruits and vegetables (g/day)	308.0 (155.6)	156	1.02 (0.97–1.07)
Free sugars (en%/day)^3^	6.6 (4.3)	4	1.03 (0.98–1.08)

1 The increment is a rounded number close to the s.d. of the component.

2 All models were stratified cohort, and adjusted for sex, age at baseline, body mass index, smoking status, education, physical activity, energy intake without energy from alcohol, and alcohol intake.

3 (en%) refers to the percentage of total energy intake excluding alcohol.

Abbreviations: s.d., standard deviation, m, milligrams, g, grams, en%, energy percentage, HR, hazard ratio, CI, confidence interval.

To address potential residual confounding by tobacco smoking, we repeated the main analysis in never smokers and found results comparable to results in the total population after adjusting for smoking (HR of overall cancer associated with a one-point increment of the HDI was 0.99 (95% CI 0.94–1.05)).

Excluding cancers occurring within two years of follow-up did not alter the association with overall cancer (data not shown). Re-analysing the data replacing the current HDI with the HDI based on the 1990 WHO guidelines resulted in risk estimates of the same order of magnitude [10].

## Discussion

This study shows that higher adherence to the WHO’s Healthy Diet Indicator, a dietary pattern for prevention of chronic diseases, was not associated with overall, smoking- or alcohol-related cancer. Each point increment of the HDI reduced risk in men by 4%, but this was statistically not significant, while the association was null in women. In additional sensitivity analyses, estimates for overall cancer risk proved relatively robust.

Two previous studies [10], [11] related the HDI, based on the WHO guidelines of 1990, to overall mortality but not cancer risk; therefore we could not directly compare results. Both studies did find inverse associations with overall-mortality in different pooled populations. Huijbregts et al. found a reduction in overall mortality of 13% for people with highest versus lowest tertile of HDI (95% CI: 2–23%) [10]. The reported estimate for the Netherlands was 25%, but not statistically significant, possibly due to lack of power. The difference in associations could be due to the population selection: Huijbregts et al studied only elderly men, while we also found lower risk estimates in men. However our power in males was limited due to low numbers of male cases (586 versus 1796 in Huijbregts’ study). In addition, men in our study were quite young at the end of follow up, and dietary patterns at older ages may differ. In the study of Knoops et al., estimates for individual countries were not provided, however the estimate for Northern Europe was comparable with our estimate (HR: 0.93; 95% CI: 0.85–1.02) [11]. In addition, another study found no reduction of risk of breast cancer for a higher HDI in British women (HR for maximal adherence to the HDI compared with minimal adherence: 0.94 with 95% CI: 0.67–1.32) [12].

Although the HDI has been associated with reduced all-cause mortality and reduced mortality from cardiovascular diseases, in our study this indicator was not related to cancer risk. Our results are consistent with other studies, showing that scores that include beneficial dietary components, but not other lifestyle factors such as overweight and smoking, are only weakly related to cancer risk if related at all [28]–[33]. It may be that dietary scores aiming for general prevention of chronic diseases, are more strongly associated with cardiovascular disease than with cancer because of the specific dietary components included [34]. For example, red meat and dairy products were not included in the HDI, whereas red meat is an established risk factor for colon cancer, and milk has been shown to be protective for this type of cancer [35]–[39]. There is also debate on whether dietary scores should incorporate a weight loss component, since excess body weight is directly associated with risk of cancer [40]. Although we adjusted our analyses for body mass index, it is possible that this did not completely account for the influence of body fatness. This is supported by two recent studies, showing that a diet-lifestyle score, including the component body fatness, was significantly associated with cancer risk [41], [42].

An alternative explanation for our null results is that for some dietary components, the range of intake from low to high HDI adherence in our study population was relatively modest, which could be an indication for small between-person variance in diet in relation to within-person variance. Furthermore, all seven components, and related foods have been equally weighted in the computation of the HDI although their associations with cancer may differ. Creating a priori dietary scores like the HDI requires researchers to make arbitrary decisions concerning the foods or nutrients to be included, their scoring, and the cut-off values to be used [6]. A posteriori methods could overcome these issues by grouping participants according to their dietary characteristics and similarity.

Advantages of the present study are the prospective design, the long follow-up period and complete ascertainment of cancers, the large sample size specific for women and the inclusion of a number of potential important confounders. In addition to overall cancer, we studied cancer sites specifically related to alcohol and smoking. We adjusted for study cohort (i.e. Prospect or Morgen) to adjust for differences in study population.

There were several limitations to this study. Although the FFQ used in this study had been validated, results could have been affected by measurement error in dietary intake. Particularly for fat and protein intake, correlations with intakes obtained through 24-h recalls were modest (fat; men: 0.61; women: 0.63 and protein; men: 0.71; women: 0.67) [18], [19]. Moreover, correlations for saturated fat, poly-unsaturated fatty acids, or cholesterol (all used for construction of the HDI) were not available from the validation study. A potential limitation may be that cases could have modified their diet during the early pre-diagnostic period; however, excluding incident cases diagnosed in the first 2 years of follow-up did not alter associations. Underreporting by participants with high energy or fat intakes could also have played a role; especially in women since they are more likely than men to underreport their intake [43].

Physical activity was missing for 14% of the EPIC-NL cohort. Simply excluding these participants would have provided biased results, since missing data did not occur completely at random and this may have resulted in misclassification of physical activity for the concerned participants [44]. We therefore imputed these missing values using single imputation. Women in Prospect (approximately 59% of our female study population) were participating in a screening trial, and this could be associated with healthier (dietary) behaviours. This may limit generalizability of our results to women exposing more unhealthy behaviour. However, still a major part of our study population (women and men within the MORGEN cohort) was reflecting the general Dutch population. More importantly, the fact that women within our study may have been altogether slightly more healthy does not affect the internal validity of our study. Prevalence estimates of baseline characteristics might have been more favourable, but this does not cause bias in the examined associations, as was demonstrated in a previous study using data from the Morgen cohort [45].

The possibility of residual confounding in the present study cannot be ruled out, although we were able to control for important factors as smoking, level of education, physical activity and anthropometric indicators. When we repeated the main analysis in never-smokers, estimates did not change notably. It is possible that the baseline dietary measurement became increasingly irrelevant to the development of cancers arising later after baseline because of altered dietary habits. However, when we performed an analysis by different follow-up periods, associations for overall cancer were comparable for all follow-up periods.

In conclusion, in this population-based prospective cohort study, a healthy diet defined by WHO guidelines was not associated with overall cancer risk in men or women. The components of the HDI may be too broad to detect an association with cancer. Future studies investigating diet and cancer risk should take into account other lifestyle components besides a healthy diet.

## References

[1] Ferlay J, Shin HR, Bray F, Forman D, Mathers C, et al (2010) Estimates of worldwide burden of cancer in 2008: GLOBOCAN 2008. Int J Cancer 127: 2893–2917. 21351269 10.1002/ijc.25516

[2] Statistics Netherlands (2011): CBS.

[3] Ross SA (2010) Evidence for the relationship between diet and cancer. Exp Oncol 32: 137–142. 21403607

[4] Anand P, Kunnumakkara AB, Sundaram C, Harikumar KB, Tharakan ST, et al (2008) Cancer is a preventable disease that requires major lifestyle changes. Pharm Res 25: 2097–2116. 18626751 10.1007/s11095-008-9661-9 PMC2515569

[5] World Cancer Research Fund, American Institute for Cancer Research (WCRF/AICR) Cancer preventability estimates for food, nutrition, body fatness, and physical activity. Continuous Update Project Washington, DC: AICR. Available: http://www.wcrf.org/cancer_statistics/preventability_estimates/preventability_estimates_food.php. Accessed 12 April 2013.

[6] Waijers PM, Feskens EJ, Ocke MC (2007) A critical review of predefined diet quality scores. Br J Nutr 97: 219–231. 17298689 10.1017/S0007114507250421

[7] Kant AK (2004) Dietary patterns and health outcomes. J Am Diet Assoc 104: 615–635. 15054348 10.1016/j.jada.2004.01.010

[8] Couto E, Boffetta P, Lagiou P, Ferrari P, Buckland G, et al (2011) Mediterranean dietary pattern and cancer risk in the EPIC cohort. Br J Cancer Apr 26 104(9): 1493–9. 10.1038/bjc.2011.106 PMC3101925 21468044

[9] Verberne L, Bach-Faig A, Buckland G, Serra-Majem L (2010) Association between the Mediterranean diet and cancer risk: a review of observational studies. Nutr Cancer 62: 860–870. 20924961 10.1080/01635581.2010.509834

[10] Huijbregts P, Feskens E, Rasanen L, Fidanza F, Nissinen A, et al (1997) Dietary pattern and 20 year mortality in elderly men in Finland, Italy, and The Netherlands: longitudinal cohort study. BMJ 315: 13–17. 9233319 10.1136/bmj.315.7099.13 PMC2127011

[11] Knoops KT, Groot de LC, Fidanza F, Alberti-Fidanza A, Kromhout D, et al (2006) Comparison of three different dietary scores in relation to 10-year mortality in elderly European subjects: the HALE project. Eur J Clin Nutr 60: 746–755. 16418742 10.1038/sj.ejcn.1602378

[12] Cade JE, Taylor EF, Burley VJ, Greenwood DC (2011) Does the Mediterranean dietary pattern or the Healthy Diet Index influence the risk of breast cancer in a large British cohort of women? Eur J Clin Nutr 65: 920–928. 21587285 10.1038/ejcn.2011.69

[13] Riboli E, Kaaks R (1997) The EPIC Project: rationale and study design. European Prospective Investigation into Cancer and Nutrition. Int J Epidemiol 26 Suppl 1S6–14. 9126529 10.1093/ije/26.suppl_1.s6

[14] Beulens JW, Monninkhof EM, Verschuren WM, van der Schouw YT, Smit J, et al (2009) Cohort profile: the EPIC-NL study. Int J Epidemiol 39: 1170–1178. 19483199 10.1093/ije/dyp217

[15] Boker LK, van Noord PA, van der Schouw YT, Koot NV, Bueno de Mesquita HB, et al (2001) Prospect-EPIC Utrecht: study design and characteristics of the cohort population. European Prospective Investigation into Cancer and Nutrition. Eur J Epidemiol 17: 1047–1053. 12380720 10.1023/a:1020009325797

[16] Blokstra A, Smit HA, Verschuren WMM, Bueno de Mesquita HB, Seidell JC (1998) The monitoring project on risk factors for chronic diseases (MORGEN project) annual report 1997 (in Dutch). RIVM (National Institute for Public Health and the Environment), Bilthoven, The Netherlands.

[17] WHO (1990) Diet, nutrition, and the prevention of chronic diseases. Report of a WHO Study Group. World Health Organ Tech Rep Ser 797: 1–204. 2124402

[18] Ocke MC, Bueno-de-Mesquita HB, Pols MA, Smit HA, van Staveren WA, et al (1997) The Dutch EPIC food frequency questionnaire. II. Relative validity and reproducibility for nutrients. Int J Epidemiol 26 Suppl 1S49–58. 9126533 10.1093/ije/26.suppl_1.s49

[19] Ocke MC, Bueno-de-Mesquita HB, Goddijn HE, Jansen A, Pols MA, et al (1997) The Dutch EPIC food frequency questionnaire. I. Description of the questionnaire, and relative validity and reproducibility for food groups. Int J Epidemiol 26 Suppl 1S37–48. 9126532 10.1093/ije/26.suppl_1.s37

[20] WHO (2003) Diet, nutrition, and the prevention of chronic diseases. Report of a WHO Study Group. World Health Organ Tech Rep Ser 916: 1–149. 12768890

[21] International Agency for Research on Cancer (IARC) Working Group on the Evaluation of Carcinogenic Risks to Humans (2004) Tobacco smoke and involuntary smoking. Monogr Eval Carcinog Risks Hum 83. PMC4781536 15285078

[22] Secretan B, Straif K, Baan R, Grosse Y, El Ghissassi F, et al (2009) A review of human carcinogens–Part E: tobacco, areca nut, alcohol, coal smoke, and salted fish. Lancet Oncol 10: 1033–1034. 19891056 10.1016/s1470-2045(09)70326-2

[23] Boffetta P, Couto E, Wichmann J, Ferrari P, Trichopoulos D, et al (2010) Fruit and vegetable intake and overall cancer risk in the European Prospective Investigation into Cancer and Nutrition (EPIC). J Natl Cancer Inst 102: 529–537. 20371762 10.1093/jnci/djq072

[24] Haftenberger M, Schuit AJ, Tormo MJ, Boeing H, Wareham N, et al (2002) Physical activity of subjects aged 50–64 years involved in the European Prospective Investigation into Cancer and Nutrition (EPIC). Public Health Nutr 5: 1163–1176. 12639225 10.1079/PHN2002397

[25] Wareham NJ, Jakes RW, Rennie KL, Schuit J, Mitchell J, et al (2003) Validity and repeatability of a simple index derived from the short physical activity questionnaire used in the European Prospective Investigation into Cancer and Nutrition (EPIC) study. Public Health Nutr 6: 407–413. 12795830 10.1079/PHN2002439

[26] InterAct Consortium (2012) Validity of a short questionnaire to assess physical activity in 10 European countries. Eur J Epidemiol 27: 15–25. 22089423 10.1007/s10654-011-9625-y PMC3292724

[27] Sluijs I, van der Schouw YT, van der AD, Spijkerman AM, Hu FB, et al (2010) Carbohydrate quantity and quality and risk of type 2 diabetes in the European Prospective Investigation into Cancer and Nutrition-Netherlands (EPIC-NL) study. Am J Clin Nutr 92: 905–911. 20685945 10.3945/ajcn.2010.29620

[28] Cerhan JR, Potter JD, Gilmore JM, Janney CA, Kushi LH, et al (2004) Adherence to the AICR cancer prevention recommendations and subsequent morbidity and mortality in the Iowa Women’s Health Study cohort. Cancer Epidemiol Biomarkers Prev 13: 1114–1120. 15247121

[29] Fitzgerald AL, Dewar RA, Veugelers PJ (2002) Diet quality and cancer incidence in Nova Scotia, Canada. Nutr Cancer 43: 127–132. 12588692 10.1207/S15327914NC432_2

[30] Harnack L, Nicodemus K, Jacobs DR Jr, Folsom AR (2002) An evaluation of the Dietary Guidelines for Americans in relation to cancer occurrence. Am J Clin Nutr 76: 889–896. 12324305 10.1093/ajcn/76.4.889

[31] McCullough ML, Feskanich D, Rimm EB, Giovannucci EL, Ascherio A, et al (2000) Adherence to the Dietary Guidelines for Americans and risk of major chronic disease in men. Am J Clin Nutr 72: 1223–1231. 11063453 10.1093/ajcn/72.5.1223

[32] McCullough ML, Feskanich D, Stampfer MJ, Giovannucci EL, Rimm EB, et al (2002) Diet quality and major chronic disease risk in men and women: moving toward improved dietary guidance. Am J Clin Nutr 76: 1261–1271. 12450892 10.1093/ajcn/76.6.1261

[33] McCullough ML, Feskanich D, Stampfer MJ, Rosner BA, Hu FB, et al (2000) Adherence to the Dietary Guidelines for Americans and risk of major chronic disease in women. Am J Clin Nutr 72: 1214–1222. 11063452 10.1093/ajcn/72.5.1214

[34] McCullough ML, Giovannucci EL (2004) Diet and cancer prevention. Oncogene 23: 6349–6364. 15322510 10.1038/sj.onc.1207716

[35] Chan AT, Giovannucci EL (2010) Primary prevention of colorectal cancer. Gastroenterology 138: 2029–2043 e2010. 10.1053/j.gastro.2010.01.057 PMC2947820 20420944

[36] Chan DS, Lau R, Aune D, Vieira R, Greenwood DC, et al (2011) Red and processed meat and colorectal cancer incidence: meta-analysis of prospective studies. PLoS One 6: e20456. 21674008 10.1371/journal.pone.0020456 PMC3108955

[37] Huncharek M, Muscat J, Kupelnick B (2009) Colorectal cancer risk and dietary intake of calcium, vitamin D, and dairy products: a meta-analysis of 26,335 cases from 60 observational studies. Nutr Cancer 61: 47–69. 19116875 10.1080/01635580802395733

[38] Pufulete M (2008) Intake of dairy products and risk of colorectal neoplasia. Nutr Res Rev 21: 56–67. 19079854 10.1017/S0954422408035920

[39] Aune D, Lau R, Chan DS, Vieira R, Greenwood DC, et al (2012) Dairy products and colorectal cancer risk: a systematic review and meta-analysis of cohort studies. Ann Oncol 23: 37–45. 21617020 10.1093/annonc/mdr269

[40] Bianchini F, Kaaks R, Vainio H (2002) Overweight, obesity, and cancer risk. Lancet Oncol 3: 565–574. 12217794 10.1016/s1470-2045(02)00849-5

[41] Vergnaud AC, Romaguera D, Peeters PH, van Gils CH, Chan DS, et al.. (2013) Adherence to the World Cancer Research Fund/American Institute for Cancer Research guidelines and risk of death in Europe: results from the European Prospective Investigation into Nutrition and Cancer cohort study. Am J Clin Nutr. 10.3945/ajcn.112.049569 23553166

[42] Romaguera D, Vergnaud AC, Peeters PH, van Gils CH, Chan DS, et al (2012) Is concordance with World Cancer Research Fund/American Institute for Cancer Research guidelines for cancer prevention related to subsequent risk of cancer? Results from the EPIC study. Am J Clin Nutr 96: 150–163. 22592101 10.3945/ajcn.111.031674

[43] Klesges RC, Eck LH, Ray JW (1995) Who underreports dietary intake in a dietary recall? Evidence from the Second National Health and Nutrition Examination Survey. J Consult Clin Psychol 63: 438–444. 7608356 10.1037//0022-006x.63.3.438

[44] Donders AR, van der Heijden GJ, Stijnen T, Moons KG (2006) Review: a gentle introduction to imputation of missing values. J Clin Epidemiol 59: 1087–1091. 16980149 10.1016/j.jclinepi.2006.01.014

[45] Van Loon AJ, Tijhuis M, Picavet HS, Surtees PG, Ormel J (2003) Survey non-response in the Netherlands: effects on prevalence estimates and associations. Ann Epidemiol 13: 105–110. 12559669 10.1016/s1047-2797(02)00257-0

